# Induction and Genome Analysis of HY01, a Newly Reported Prophage from an Emerging Shrimp Pathogen *Vibrio campbellii*

**DOI:** 10.3390/microorganisms9020400

**Published:** 2021-02-15

**Authors:** Taiyeebah Nuidate, Aphiwat Kuaphiriyakul, Komwit Surachat, Pimonsri Mittraparp-arthorn

**Affiliations:** 1 Division of Biological Science, Faculty of Science, Prince of Songkla University, Hat Yai, Songkhla 90110, Thailand; milk.nootai@gmail.com (T.N.); golden_2468@hotmail.com (A.K.); 2 Division of Computational Science, Faculty of Science, Prince of Songkla University, Hat Yai, Songkhla 90110, Thailand; komwit.s@psu.ac.th; 3 Molecular Evolution and Computational Biology Research Unit, Faculty of Science, Prince of Songkla University, Hat Yai, Songkhla 90110, Thailand

**Keywords:** CRISPR, luminous vibriosis, prophage, *Siphoviridae*, *Vibrio campbellii*

## Abstract

*Vibrio campbellii* is an emerging aquaculture pathogen that causes luminous vibriosis in farmed shrimp. Although prophages in various aquaculture pathogens have been widely reported, there is still limited knowledge regarding prophages in the genome of pathogenic *V. campbellii*. Here, we describe the full-genome sequence of a prophage named HY01, induced from the emerging shrimp pathogen *V. campbellii* HY01. The phage HY01 was induced by mitomycin C and was morphologically characterized as long tailed phage. *V. campbellii* phage HY01 is composed of 41,772 bp of dsDNA with a G+C content of 47.45%. A total of 60 open reading frames (ORFs) were identified, of which 31 could be predicted for their biological functions. Twenty seven out of 31 predicted protein coding regions were matched with several encoded proteins of various *Enterobacteriaceae*, *Pseudomonadaceae*, *Vibrionaceae*, and other phages of Gram-negative bacteria. Interestingly, the comparative genome analysis revealed that the phage HY01 was only distantly related to Vibrio phage Va_PF430-3_p42 of fish pathogen *V. anguillarum* but differed in genomic size and gene organization. The phylogenetic tree placed the phage together with *Siphoviridae* family. Additionally, a survey of Clustered Regularly Interspaced Short Palindromic Repeats (CRISPR) spacers revealed two matching sequences between phage HY01 genome and viral spacer sequence of *Vibrio* spp. The spacer results combined with the synteny results suggest that the evolution of *V. campbellii* phage HY01 is driven by the horizontal genetic exchange between bacterial families belonging to the class of Gammaproteobacteria.

## 1. Introduction

*Vibrio campbellii* is a Gram-negative bacterium belonging to the family *Vibrionaceae* and is widely distributed in the marine environment [1]. It is one of the major pathogens in farmed shrimp that causes luminous vibriosis, which leads to huge economic losses worldwide. *V. campbellii* HY01 (previously known as *V. harveyi* HY01) was isolated from a dead shrimp during luminous vibriosis outbreak in southern Thailand [2]. This strain also exhibited high virulence causing 100% mortality in shrimp after 12 h of injection. Moreover, it contained the *hhl* hemolysin gene, a homolog of *hlyA* gene of *V. cholerae*, which might be derived from horizontal gene transfer (HGT) [3].

Temperate phages (i.e., phages that integrate their genome into their host’s chromosome entering the dormant stated known as prophage) are important agents of HGT. As prophages, temperate phages can contribute to their bacterial host’s pathogenesis and enhance their host’s fitness [4]. Phages infecting *V. cholerae* (vibriophages) can mediate horizontal transfer of the clusters of genes. Additionally, both the genomic rearrangements and bactericidal selection of the host are also mediated by vibriophages [5]. The interplay between the most of vibriophages and prophages with CTXφ phage can promote the horizontal transfer the cholera toxin genes [6]. In a study by Castillo et al., 28 out of 64 of *Vibrio* species carried a complete prophage-like element that encodes the zonula occludens toxin (Zot), the cytotoxin first detected in *V. cholerae* [7]. *V. campbellii* strain 642 contains the phage *V. harveyi* myovirus (VHML), which is associated with an increased virulence and resistance to the vibriostatic compound O/129 (150 µg) [8,9].

Although more rarely, lytic phages can also disseminate virulence genes via HGT [10], the transfer of CTXɸ genes from *V. cholerae* O1 El Tor strain to non-O1/O139 strain was mediated by lytic phages, which indicates that transduction is one of the possible mechanisms of pathogenic evolution among surrounding bacteria [11]. In addition, several lytic phages against *Vibrio* species have been described [12,13,14]. The presumably lytic phage OKB54 specific for *V. campbellii* was efficiently targeting five tested strains [15], while phage P4A and P4F belonging to the family *Siphoviridae* were able to lyse *Vibrio* strain BF4, which belongs to the Harveyi clade of the genus *Vibrio* [16]. Yet, little is known about the induction and characterization of pathogenic *V. campbellii* prophage. To the best of our knowledge, only one study described the genomic characteristics of prophages from pathogenic *V. campbellii* BAA-1116 (also known as strain BB120). The prophages likely belonged to the family *Myoviridae*, were found in chromosome I and chromosome II, and were related to phage ɸHAP-1 and kappa-like *Vibrio* phage, respectively [17].

In this study, phage HY01 was isolated from *V. campbellii* HY01, a notorious shrimp pathogen by mitomycin C induction. The morphology and complete genome sequence of phage HY01 were analyzed to explain the evolutionary interaction between this phage and its host and this phage and other phages, and to gain insight into the anti-phage mechanisms of bacteria via CRISPR–Cas systems.

## 2. Materials and Methods

### 2.1. Bacterial strain and Culture Conditions

*V. campbellii* HY01 isolated from a dead shrimp with luminous vibriosis was used as a host for phage HY01. The bacterium was cultured in tryptic soy agar containing 1% *w*/*v* NaCl and incubated at 30 °C for 16–18 h. A single colony of bacteria was transferred to 40 mL LB-MOPS (10 g/L NaCl, 10 g/L tryptone, 5 g/L yeast extract, and 10.5 g/L MOPS) medium with 1% *w*/*v* NaCl and further incubated with shaking (150 rpm) at 30 °C for 4–6 h. A logarithmic phase culture (OD 600 nm = 0.4–0.6) of *V. campbellii* HY01 was used for the prophage induction experiment.

### 2.2. Prophage Induction Using Mitomycin C, Heat Treatment, and UV Radiation

The prophage was induced with mitomycin C according to Lorenz et al. [17] with slight modifications. Briefly, *V. campbellii* was cultured in LB medium. Mitomycin C (Sigma-Aldrich, St Louis, USA) at the final concentration of 1 µg/mL was added to the culture of *V. campbellii* HY01 during logarithmic phase in 12 mL LB medium and incubated for 30 min. The bacterial cells were collected by centrifugation at 10,000× *g* for 10 min, washed twice with fresh LB medium, and resuspended in 12 mL LB medium. Bacterial lysis was observed after 2 h incubation at 30 °C by measuring the optical density at 600 nm using a spectrophotometric microplate reader (LUMIstar Omega, BMG Labtech, Ortenberg, Germany). Untreated bacterial cells were used as a control. The presence of induced phage was confirmed with double agar overlay plaque assay at 30 °C. In addition, heat treatment and UV irradiation were used to induce *V. campbellii* HY01. The bacterial cells were heated at 50 °C for 30 min, then subsequently transferred to 30 °C and the optical density measured at 600 nm. For UV irradiation, the prophage was induced according to Jäckel et al. [18] with some modifications. Briefly, the bacterial cells during logarithmic phase were exposed to UV irradiation by applying aliquots of the culture into petri dishes and placing those in a distance of 10 cm to an UV lamp (corresponding 45 J m−2) for 10 min. Non-UV irradiated bacterial cells were used as a control. The presence of induced phage was observed with double agar overlay plaque assay. All experiments were carried out in triplicate.

### 2.3. Preparation of Concentrated Phage

One hundred milliliters of mitomycin-C-induced cell were centrifuged at 10,000× *g* for 10 min. To eliminate the bacterial cell debris and intact cells, the supernatant was filtered through a 0.2 µm filter. The filtrate supernatant was mixed with polyethylene glycol 8000 (PEG8000) at a final concentration of 100 g/L and incubated overnight at 4 °C. Centrifugation using an Optima LE-80K ultracentrifuge (Beckman Coulter, USA) at 25,000× *g* for 3 h at 4 °C was operated to collect the phage particles. The collected pellet was dissolved in 0.5 mL SM buffer (10mM NaCl, 50 mM Tris/HCl (pH 7.5), 10 mM MgSO4) and stored at 4 °C for further applications.

### 2.4. Transmission Electron Microscopy (TEM)

Phage morphology was examined by dropping 10 µL of purified phage lysate at a concentration of 10^8^ PFU/mL onto formvar carbon coated grids (Electron Microscopy Sciences, Hatfield, PA, USA) and stained with 2% uranyl acetate for 5 min [19]. The negative staining grid was examined using a transmission electron microscope, JEM-100CX II (Jeol, Tokyo, Japan) at 80 kV with magnification of 80,000x. 

### 2.5. Phage DNA Extraction

DNA extraction was performed by incubating 1 mL of purified phage lysate (10^10^ PFU/mL) with DNase I (final concentration 1 ug/mL) and RNase A (final concentration 30 ug/mL) to remove host nucleic acid contamination. After 30 min of incubation at room temperature, the mixture was heated at 75 °C for 5 min to inactivate DNase I. Then, the mixture was treated with 4 µL of proteinase K (20 mg/mL) and lysis buffer B (Phage DNA isolation kit) for 1 h at 56 °C. DNA was further extracted using the Phage DNA isolation kit (Norgen, Biotek, Thorold, ON, Canada) according to the manufacturer’s instructions. The extracted phage DNA was subsequently sequenced.

### 2.6. Phage DNA Assembly and Analysis

The quality of phage HY01 whole genome was evaluated using NanoDrop (Maestrogen, Inc., Las Vegas, NV, USA) followed by library construction using the TruSeq DNA PCR-Free kit (Illumina, San Diego, CA, USA). The phage was paired-end (2 × 100 bp), whole-genome sequenced using the Illumina HiSeq 2000 platform, and generated reads were assembled using SPAdes version 3.11.1 [20]. Then, the assembled contigs were annotated and open reading frames (ORFs) identified, applying various computational software in combination: the Rapid Annotation using Subsystem Technology (RAST) version 2.0 [21], GeneMarkS version 3.25, (http://exon.gatech.edu/GeneMark/gmhmmp.cgi) [22], and PHASTER (PHAge Search Tool Enhanced Release) web server, which uses NCBI’s BLAST+, version 2.3.0+ algorithm for the annotation of prophage genomes [23]. The protein was named “hypothetical” when yielded protein predictions from RAST, GeneMarkS, and PHASTER were discordant. The hypothetical protein was followed by manual verification to curate possible gene functions and to identify the prophage relatives by screening all the predicted proteins using BLASTP and PSI-BLASTP against the non-redundant (nr) NCBI database with threshold E value of 10^-4^. The tRNAscan-SE program was used to search for tRNA genes [24]. Phages closely related to phage HY01 were identified using an online BLASTn search of the NCBI database (https://blast.ncbi.nlm.nih.gov/Blast.cgi). A genomic map of phage HY01 was generated with Geneious Prime version 2020.1.2 [25]. The CRISPR/Cas loci in *V. campbellii* HY01 genome and any CRISPR spacers in the phage HY01 genome were examined by BLAST against the CRISPR database using the BLAST CRISPRs function (https://crispr.i2bc.paris-saclay.fr/crispr/) and positional searching using the Integrated Microbial Genome/Virus (IMG/VR) viral spacer database (https://img.jgi.doe.gov/cgi-bin/vr/main/cgi) [26]. Additionally, the spacer sequences were identified by submitting phage HY01 sequence to BLASTN (somewhat similar sequences) with the Entrez Query “Vibrio”.

### 2.7. Phylogenetic Analysis 

The sequences of some “core” genes encoding major capsid protein, the large terminase protein subunit, portal protein, lysozyme, and tailed protein that were demonstrated to be useful for phylogenetic analysis of phages were used to examine the phylogenetic position of phage HY01 at the viral tree of life. The amino acid sequences of phage HY01 and other related phages with genome-wide nucleotide similarities to HY01 as computed using BLASTN were aligned using ClustalW with the default parameters [27], and the phylogenetic trees were generated using the neighbor-joining method [28] with 1000 bootstrap replicates in MEGA-X version 10.0.5 [29]. In addition, the ViPTree was used to find the closest relative of phage HY01 by constructing a viral proteomic tree [30].

### 2.8. Accession Numbers of Phage HY01 Genome

The genome sequence of phage HY01 was submitted to the NCBI database under the accession number MT366580.

### 2.9. Statistical Analysis

Two-way ANOVA was performed for statistical analysis using SPSS software version 14 (SPSS inc., Chicago, IL, USA). The *p* value of < 0.05 was considered a statistically significant result.

## 3. Results

### 3.1. Induction of Prophage HY01

**Figure 1:**
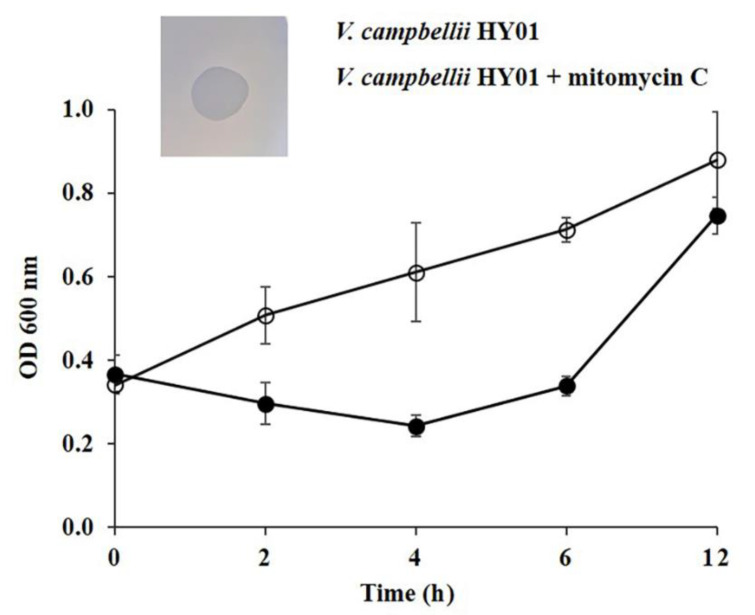
Growth rate of *V. campbellii* HY01 after induction of prophage with mitomycin C. Induced culture is marked with filled circle (●) and non-induced culture with empty circle (○). *V. campbellii* HY01 showed a decrease in turbidity 2 h after mitomycin C induction (1 µg/mL). The inserted photograph shows a double agar overlay plaque assay of the induced phage on *V. campbellii* HY01 after mitomycin C induction.

Phages obtained from *V. campbellii* HY01 were induced with mitomycin C. The logarithmic phase culture of *V. campbellii* HY01 was incubated with mitomycin C (1 ug/mL) for 30 min, washed twice, and further incubated. Bacterial cell density after mitomycin C treatment for 4–6 h was significantly decreased compared to the untreated control (ANOVA *n* = 3, *p* < 0.05) (Figure 1 and Table S1). This might have been caused by the release of inducible prophages that led to bacterial cell lysis [31]. To determine whether the decreasing in optical density was indeed caused by bacterial cell lysis, the bacterial supernatant was dropped on the *V. campbellii* HY01 lawn. A turbid plaque was observed, consistent with the nature of prophage, indicating a high frequency of lysogenization. In contrast, both heat treatment and UV irradiation were not capable of inducing prophage from *V. campbellii* HY01 (data not shown). This might be due to unsuitable induction times and conditions. Therefore, the optimum condition for heat treatment and UV irradiation should be evaluated. 

### 3.2. Transmission Electron Micrographs of Phage Particle

**Figure 2:**
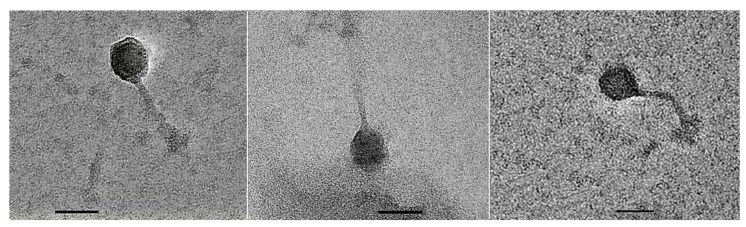
Representative transmission electron microscopy (TEM) image of mitomycin C induced phage HY01 recovered from the supernatant of *V. campbellii* HY01. The phage was negatively stained with 2% uranyl acetate. The scale bar represents 50 nm.

The induced phage was named HY01. The morphology of phage HY01 was examined by transmission electron microscopy (TEM). The phage HY01 had an icosahedral capsid with a diameter of approximately 45 nm and long non-contractile tail of approximately 100 nm (Figure 2). The phage HY01 probably belongs to the Siphovirus morphotype, as a long tail was observed, similar to other *Siphoviridae* phages [32]. 

### 3.3. Phage HY01 Genome and Comparative Genomic Analysis

Following assemblies with SPAdes, BLASTN alignments showed that the sequence of phage HY01 was highly homologous to the location of 145 to 41,916 bp of *V. campbellii* HY01 genome with 100% nucleotide identity (Table S1). The genome of phage HY01 contains double-stranded DNA with 41,772 base pairs (bp) in length and a G+C content of 47.45% (Table 1), which is closely related to that of host *V. campbellii* HY01 genome (45.50%). This finding is consistent with previously reported G+C contents of prophages and their host bacterium. One tRNA sequence found in phage HY01 genome (ORF 33) was considered as a factor to promote phage integration in the host genome by improving the efficiency of phage protein translation and thus expediting phage replication [33]. Additionally, antibiotic resistance genes or bacterial virulence genes were not found in the genome of phage HY01. 

The nucleotide sequence of phage HY01 was searched with BLASTN against phage genomes in NCBI database. The genomic sequence of phage HY01 genome had the highest similarity to that of Vibrio phage Va_PF430-3_p42 (NCBI:MK672805.1) isolated from fish pathogen *Vibrio anguillarum* with 70.68% identity and 3% query coverage (Table S2). Overall, there was a low percentage of coverage between phage HY01 and other related phages (1–2% coverage). 

### 3.4. ORFs Analysis and Comparative Proteomic Analysis of Phage HY01

The open reading frames (ORFs) of the putative phage HY01 genome were investigated by a combination of RAST annotation, GenemarkS, and PHASTER programs. BLASTP and PSI-BLASTP against the NCBI non-redundant protein database were used to assign the functions of identified protein coding genes (Table 2). The phage HY01 genome contains 60 ORFs with a size ranging from 85–3630 bp. Among the 60 ORFs of phage HY01, 51 ORFs were located at the plus strand, and nine remaining ORFs were found at the minus strand. Most of the predicted ORFs started with ATG, except for ORF11 (GTG), ORF15 (TTG), and ORF30 (TTG). For 31 ORFs (51.66%), the specific functions were predicted based on BLASTP sequence identity to other related phage proteins in the NCBI database. The remaining 29 ORFs showed no similarity with any other phage proteins, or they were predicted to encode a hypothetical protein. These results confirm the novelty of phage HY01. The phage proteins showed homology with database entries at 25.77% identity and 34–100% coverage.

**Figure 3:**
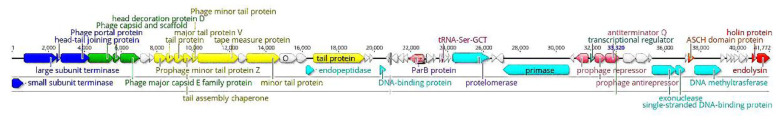
Genomic map of phage HY01. Open reading frames (ORFs) with BLASTP and PSI-BLASTP against the non-redundant (nr) NCBI database. The phage HY01 genome is illustrated using arrow symbol indicates the direction of transcription. Gene features are color-coded according to their biological functions (blue: DNA packaging; green: head structural components and assembly; yellow: tail structural components and assembly; light blue: DNA replication, modulation, and repair; rose: lysogeny control; red: lysis module). Genes coding for hypothetical proteins or unknown function are shown in light grey. The genomic map was generated using the Geneious Prime software.

Phages require various genes to achieve successful host infection including gene expression, gene regulation, DNA replication, phage capsid formation, and release of new phage particles by bacterial host lysis [34,35]. The genome of phage HY01 is divided into biological modules, which displayed a functional gene cluster for DNA packaging, head structural component and assembly, tail structural component and assembly, lysogeny control, DNA replication and modulation, and cell lysis (Figure 3). 

#### 3.4.1. DNA Packaging

DNA packaging is characteristic of double-stranded DNA (dsDNA) viruses including phage [36]. This mechanism is composed of two key components including the portal protein, which affects the procapsid assembly and provides an interface for tail attachment or assembly, and the terminase influencing the phage genome packing into the phage head. The portal protein was encoded by ORF4, which showed significant similarity to phage portal protein of various *Escherichia virus Lambda* with 55% identity. Moreover, the terminase proteins in phage HY01 genome were encoded by ORF1 and ORF2, which showed significant similarity (40.48% and 38.47% identity, respectively) to small and large subunits in the terminase of Aeromonas phage phiARM81ld (ALN97521.1). ORF3 was identified as a head–tail joining protein of phage HY01, which was found to share 48.48% identity with the head–tail joining protein of phage *Escherichia virus Lambda* (NP-040582.1).

#### 3.4.2. Head Structural Components and Assembly

The ORF5 to ORF9 are part of the head structural components and assembly modules of phage HY01. The predicted product of ORF5 was identified as phage capsid and scaffold, as it shared 45.90% identity with that of *Escherichia virus Lambda* (VUF53141.1). A head decoration protein D (ORF6) was found to share 40.96% identity with Aeromonas phage phiARM81ld (ALN97526.1), while ORF7 shared identity (42.52%) with phage major capsid E family protein of Escherichia phage YDC107_1 (AUO37489.1) and phage major capsid protein of *Escherichia virus Lambda* with 40–42% identity. No protein revealed significant similarity to ORF8 and ORF9.

#### 3.4.3. Tail Structural Components and Assembly

The functional module for tail structural component and assembly covers ORF10 to ORF21. Based on the BLASTP and PSI-BLAST similarity, ORF10, ORF14, and ORF17 were found to function as a prophage minor tail protein with *Escherichia virus N15* (NP_046906.1), Escherichia phage mEp460_ev081 (VUF53221.1), and Arthrobacter phage Ingrid (QFG08696.1), respectively. The predicted ORF 11 and ORF21 show low similarity to the tail protein in Enterobacteria phage mEp043 c-1 (YP_007111512.1) (26.53% identity) and Pseudomonas phage PS-1 (YP_009222845.1) (32.24% identity), respectively. The predicted product of ORF12 was determined as major tail protein V, because it shared 48–51%identity with various Enterobacteria phage and Escherichia phage. All tailed phages carry a gene encoding a tape measure protein (TMP) for DNA injection into their host. The length of the corresponding gene is proportional to the length of the phage tail [37]. Therefore, the PSI-BLAST analysis predicted the ORF15 to encode the tail length tape-measure protein 1 and shared 27.32% identity with that of Pseudoalteromonas phage SL25 (ASU03386.1). Tail assembly chaperones (TAC) are likely essential for the morphogenesis of all long-tailed phages [38]. The coded protein of ORF 13 in phage HY01 shared considerable (27.52%) identity with that of the TAC from Enterobacteria phage phi80 (YP_007947938.1). However, ORF 16 and ORF19 showed significant similarity to hypothetical proteins, and no protein displayed significant similarity to ORF18. Many of the putative tail proteins match *Siphoviridae* phages. Additionally, the tail sheath protein presumably exclusive to *Myoviridae* was not detected in phage HY01.

#### 3.4.4. DNA Replication, Modulation, and Repair

The DNA replication module of phage HY01 was covered by ORF24, ORF 36, ORF39, ORF48, ORF49, and ORF52. ORF24 and ORF49 were determined as the putative DNA binding protein and single-stranded DNA-binding protein of Vibrio phage VHML (NP_758895.1) with 35.44% identity and Vibrio phage VD1 (AGN34167.1) with 73.21% identity, respectively. Protelomerase is responsible for maintenance of the linear plasmid-like prophages [39]. ORF 36 in phage HY01 shared considerable 34.21% identity with that of the protelomerase of Yersinia phage PY54 (CAC88681.1). ORF39 was purposed to encoded primase in phage HY01, as it shared a limited identity (36.02%) with this protein encoded by Aeromonas phage phiARM81ld (ALN97565). ORF48 was found to share 54.73% identity with putative exonuclease of Vibrio phage 1.205.O._10N.222.51.A7 (AUR95306.1). In addition, ORF52 was found to share 66.40% identity with DNA methyltransferase of Vibrio phage Va_PF430-3_p42 (QCW19890.1). 

#### 3.4.5. Lysogeny Control

The lysogeny control module contained a prophage repressor protein and prophage anti-repressor protein, which are expected to control the lysogenic and lytic cycles of phage HY01 [40]. The repressor protein was encoded by ORF41, which displayed 41.50% identity with prophage repressor of Enterobacterial phage mEp390 (YP_007112454.1). The anti-repressor protein was encoded by ORF43, which shared 25.77% identity with that of prophage anti-repressor in *Halomonas virus HAP1* (YP_001686774.1). Moreover, ORF44 was predicted to encode antiterminator Q protein in phage HY01, although it shared a limited 26.42% identity with this kind of protein encoded by Yersinia phage PY54 (NP_892088.1). The ORF42 was identified as putative transcriptional regulator of phage HY01 based on its homology with putative transcriptional regulator (cro analog) of Salmonella phage Fels-1 (YP_001700550.1).

Additionally, partitioning protein (ParB), encoded by *parB* gene that is associated with the stable inheritance of the prophage [41], was also found in phage HY01 genome (ORF 29), as it shared 46.70% identity with ParB protein of *Escherichia virus N15* (NP_046922.1). However, neither of ParA and ParS partition of circular DNA molecules [42] were found in the phage HY01 genome.

#### 3.4.6. The Lysis Module

Holin, the protein that permeabilizes the bacterial cell membrane, and endolysin, the cell wall hydrolyzing enzyme, are usually required for programmed host cell lysis and the release of progeny phage by dsDNA viruses [43]. The phage HY01 encoded two lysis module proteins, ORF59 and ORF60. ORF59 was predicted to be a holing, because it shared 46.94% identity with bacteriophage holin HP1 family protein in Bacteriophage APSE-7. Moreover, the product of ORF 60 was estimated to be a phage lysis protein, because it shared 50% identity with lysozyme-like domain protein encoded in Vibrio phage 1.202.O._10N.222.45.E8 (YP_009812536.1) and shared 47.47% identity with putative endolysin of *V. cholerae* phage K139 (NP_536660.1).

### 3.5. Phylogenetic Analysis of Phage HY01 with Other Related Phages

**Figure 4:**
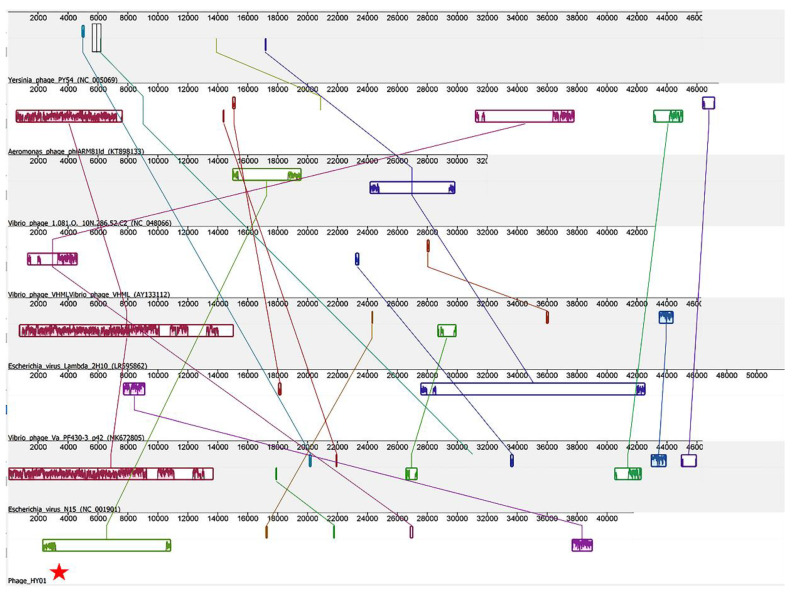
The multiple genome alignment of Gammaproteobacteria phages. Phage genomes were compared using progressiveMauve software, and homologous genomes are indicated by the connected-with-lines collinear blocks. Phage HY01 is indicated by the red star.

The nucleotide and protein sequence of phage HY01 were used to analyze the phage identity. BLASTN analysis of the phage HY01 showed low significant similarity to other sequences in the viral database (Table S2). Comparative proteomic analysis revealed that proteins encoded by phage HY01 genes were similar to proteins encoded by various phages. PSI-BLAST analysis indicated that the 27 out of 31 predicted protein shared the best identity with various Gammaproteobacteria phages, including Aeromonas phage, Escherichia phage, Enterobacteria phage, Alteromonas phage, Pseudomonas phage, Vibrio phage, Salmonella phage, and Yersinia phage (Table 2). In addition, PHASTER analysis revealed that protein encoded by phage HY01 shared 15% identity with *Escherichia virus N15* (NC_001901.1) (Table S3). The progressiveMauve alignment results showed low synteny of the phage HY01 genome with other Gammaproteobacteria phages (Figure 4). These results support the BLASTP and PSI-BLAST analysis and the biological functions shared among the genome of phage HY01 and other Gammaproteobacteria phages may suggest that the genes in phage HY01 were acquired via HGT.

**Figure 5:**
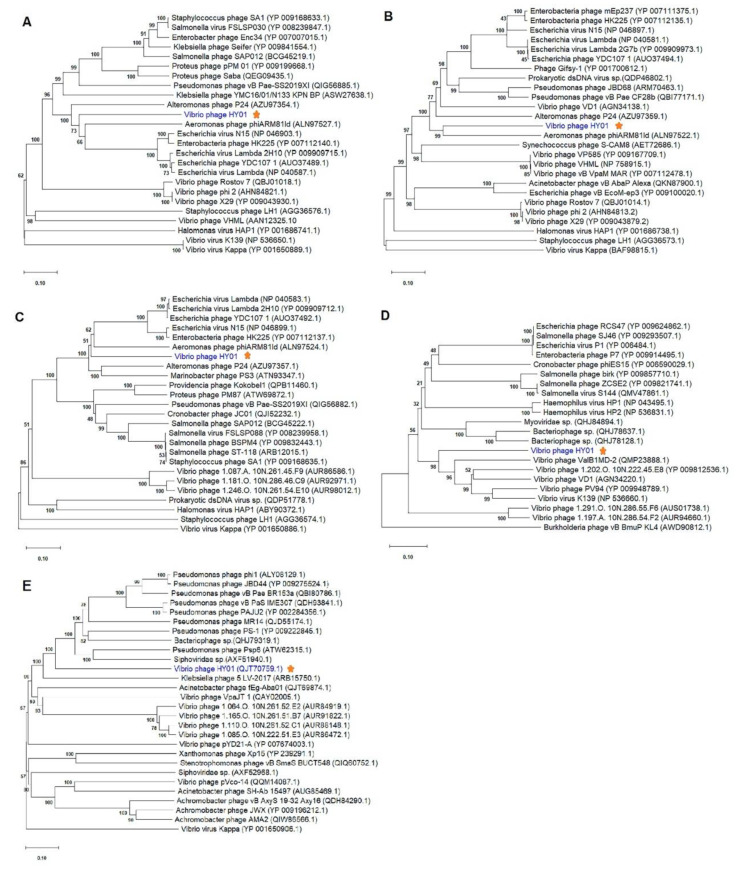
Phylogenetic analysis of phage HY01 and other related phages based on amino acid sequences of the major capsid protein (**A**), the terminase large subunit (**B**), portal protein (**C**), lysozyme (**D**), and tail protein (**E**). Bootstrap analysis was performed using the neighbor-joining method with 1000 replicates in MEGA version 10.0.5 software. The number on each node represent the support values given as percentages. The red star indicates the phage HY01.

Additionally, MEGA version 10.0.5 was applied for the phylogenetic determination of phage HY01 in order to compare phage HY01 with other phages based on amino acid sequences of the hallmark genes including major capsid protein, large terminase subunit, portal protein, lysozyme, and tail protein. The closest hit to these proteins was often a protein found in phages isolated from bacteria that belong to the *Enterobacteriaceae*. The phage HY01 proteins were phylogenetically quite distantly related to similar proteins from other phages (Figure 5). Overall, proteins from Gammaproteobacteria phages displayed the highest level of similarity with proteins of phage HY01 (Table 2 and Figure 5).

**Figure 6:**
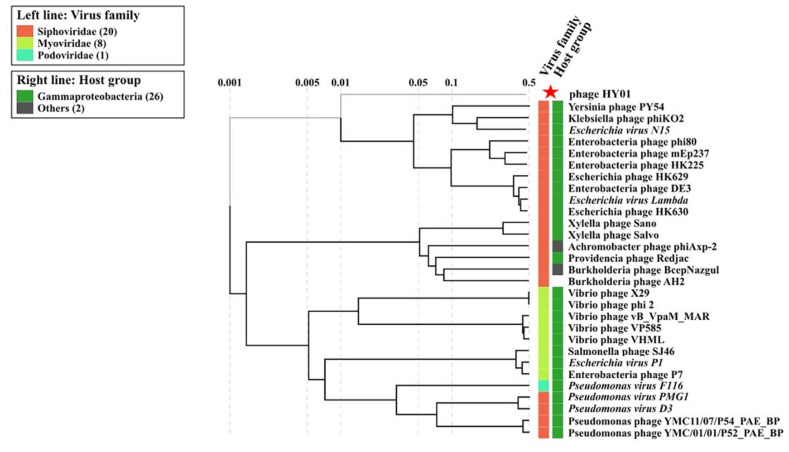
Viral proteomic tree of phage HY01 and other closely related phages. The tree was constructed using the ViPTree. The red star indicates the phage HY01.

The phylogenetic analysis based on the viral proteomic tree using ViPTree indicated phage HY01 as more closely related to Yersinia, Klebsiella, Escherichia, and Enterobacteria phages than to other known phages (Figure 6). The proteomic tree showed phage HY01 is closely related to *Siphoviridae* family. 

### 3.6. CRISPR Spacer Analysis

The existence of a CRISPR spacer identical to a phage sequence contributes to the resistance against that phage within the bacterial genomes containing that particular sequence [44]. The search for the *V. campbellii* HY01 genome against the CRISPR database showed the CRISPR/Cas loci were not detected in *V. campbellii* HY01 genome. Therefore, the genome of phage HY01 was examined for viral spacers within CRISPRs using Blast against the CRISPR database and the Viral spacer database of IMG/VR. Two matching sequences were found between phage HY01 genome and viral spacer sequences within CRISPRs. The first phage sequence that matches the spacer of *V.vulnificus* 93U204 with 96.8% identity is the AGAACCTGCAATTCCAGATTGATAACGTGACG. This sequence is found in position 15,946–15,977 bp in the phage HY01 genome. The sequence of the related spacer in *V. vulnificus* chromosome I (NZ_CP009261) is the GAACCCGCCTACAAGTGGCGGACATGCTGGAC and is found in position 511,740–511,771 bp. Additionally, this phage sequence matches 100% with various strains of *V. alginolyticus* (e.g., CP051109.1, CP017919.1, CP017889.1), 100% with two strains of *V. diabolicus* (CP042447.1, CP014036.1), 96.8% with two strains of *V. navarrensis* (CP035681.1, CP065217.1), and 93.7% with *V. natriegens* strain CCUG 16373 chromosome 1 (CP016349.1). The second phage sequence that matches the spacer of *V. parahaemolyticus* with 100% identity is the CCTATTGGACAGTTTTGGGACCGTGGACCCAG.

**Figure 7:**
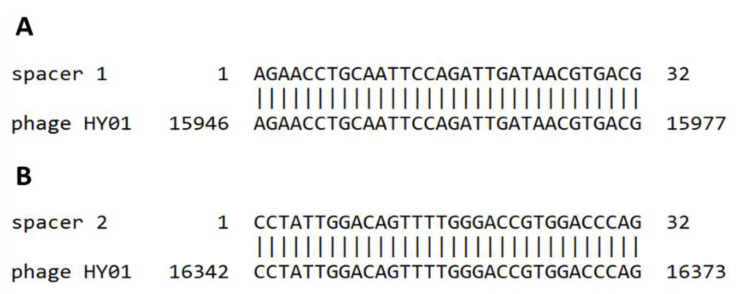
Phage HY01 genomes targeting specific within CRISPR spacers of bacteria. The complementarity between the phage HY01 sequence and spacers in various *Vibrio* spp. spacer 1 (**A**) and spacer 2 (**B**) is shown by sequence alignment.

This sequence is found in position 16,342–16,373 bp in the phage HY01 genome and as a spacer in position 1,774–1,805 bp of *V. parahaemolyticus* S141 Contig3 (accession: AWIL01000003.1). This phage sequence also yields 100% identity to *V. alginolyticus* strain 2015AW-0011 chromosome 1 (CP051109.1) and 100% identity to *V. diabolicus* strain FDAARGOS_105 chromosome 1 (CP014036.1) (Figure 7).

## 4. Discussion

Prophage-related genes may be beneficial for the pathogenicity and fitness of pathogenic bacterial strains. The knowledge of the genetic information of prophage in *V. campbellii*, a pathogenic of shrimp vibriosis, is limited. In this study, a prophage of shrimp pathogenic *V. campbellii* HY01 [3] was induced with mitomycin C, and the presence of phage was confirmed by double agar overlay plaque assay. However, heat and UV irradiation could not be induced to its lytic cycle. Previous reports showed that the prophages were induced by various methods. The mechanisms, usually based on bacterial SOS response, resulted in the inhibition of DNA replication or DNA gyrase activity [45]. Mitomycin C was found to be a more powerful inducing agent than UV radiation for the induction of *P. aeruginosa* and *Listeria* spp. prophages [46]. Different prophages may be induced by specific inducing conditions, for example, salinity, aeration, nutrient limitation, temperature, and bacterial growth rate, as various environmental factors affect the transition from lysogenic to lytic lifestyle [47,48,49]. 

This is the first report focusing on induction and genomic analysis of prophage from *V. campbellii* HY01. Morphologically characterized, the phage HY01 is a capsid phage with a long non-contractile tail and most likely belonged to the family *Siphoviridae* (Figure 2). Molecular analysis indicated that phage HY01 is a prophage. Their genome shared nucleotide and protein identities with Gammaproteobacteria phages (Table 2, Figure 4). This suggests that prophages might play a crucial role in the genetic evolution in the community of Gram-negative bacteria, especially among the family Enterobacteriaceae and Vibrionaceae.

Phage HY01 showed intermediate position between *Myo*- and *Siphoviridae* phages based on five gene markers used in phylogenetic analysis. Thus, these structural genes of phage HY01 appear to be coevolved. However, the viral proteomic tree revealed that phage HY01 had more in common with phages in the *Siphoviridae* family than it did with the phages in the *Myoviridae* family (Figure 6). We thus propose that phage HY01 is a newly reported prophage with unique sequence features that do not find the large sequence similarity to any known sequences of *Myo*- and *Siphoviridae* in the public database.

Some genes that are important for DNA packaging, head–tail structural components and assembly, and DNA replication, modulation, and repair are clustered in phage HY01 genome (Figure 3). The tRNA gene was detected as integration sites in phage HY01. These sites are frequently used by phages, genomic islands, and other mobile elements [50]. Lysogenic and lytic associated proteins encoded by phage HY01 include the prophage repressor (ORF41) and prophage anti-repressor (ORF43). These proteins are a key player in determining the life cycle of a prophage after host infection [51]. Phages employ at least two proteins, namely endolysin and holin, to coordinate a host cell lysis [52]. The phage HY01 proteins of ORF59 and ORF60 were predicted to be holin and endolysin, host cell lysis protein.

Bacteria have various mechanisms to neutralize or destroy foreign DNA including phages and plasmids. The CRISPR-Cas system is one of the most widespread of these defenses, which is a sequence-specific immunity against mobile genetic elements [44,53]. This study found two sequences matching between the phage HY01 genome and viral spacer sequence with many other marine *Vibrio* ssp. (Figure 7), suggesting past infections of these species with a closely related phage and thus tight communication trajectories among marine *Vibrio* where this or a closely related phage plays a central role [54].

Although the mechanism driving virulence is not yet clear, this work revealed central aspects of the prophage–bacteria relationship among *V. campbellii* and other *Vibrio* spp. The bacterial genomes contained prophage sequences that may affect the pathogenicity of the bacterial cell and the population fitness. Various toxin genes are phage-encoded and as a part of a diverse group of virulence factors encoded by phages [55]. Due to the low similarity of phage HY01 with existing sequenced phages, we simply do not know any genes of the identified prophage in *V. campbellii* that can be recognized as encoded bacterial toxins. However, ORF52 was putatively identified as a DNA methyltransferase, which have been reported to regulate virulence genes of many bacterial pathogens including *V. campbellii*, and the absence of this enzyme led the strains to be avirulent [9].

The absence of the CRISPR-Cas system and the presence of prophages in pathogenic *V. campbellii* HY01 might promote the acquisition of foreign genetic elements including virulence and antibiotic resistance genes and facilitate the survival of pathogens in shrimp aquaculture [56,57]. Furthermore, the properties of the phage HY01 genes encoding putative repressors could have a profound effect on the fitness of *V. campbellii* HY01 strain, not only in the aquatic environment, but also in the shrimp digestive system.

## 5. Conclusions

This is the first induction and complete genome analysis of prophage found in the genome of the shrimp pathogen *V. cambellii* HY01. The phage HY01 can be considered a new phage genus due to its nucleotide and protein levels displaying very low sequence similarity compared to other phages. The genetic information of phage HY01 plays a crucial role in the analysis of the genetic evolution of the *V. campbellii* genome. Further study requires more detection of prophage-like elements in other *V. campbellii* strains. The experimental and computational study of phage HY01 serves as important information for understanding the interactions among phages as well as among prophages and their host bacteria in the marine environment.

## Figures and Tables

**Table 1:** Phage HY01 has been identified using PHASTER.

Region	Region Length	Completeness *	Score	# Total Proteins	Region Position	Most Common Phage	GC %
1	41.7Kb	intact	110	60	1–41,772	PHAGE_Entero_N15_NC_001901(9)	47.45%

* The predicted phage associated region was defined into three scenarios according to how many genes/proteins of known phage the region contained: intact (> 90%), questionable (90–60%), and incomplete (≤ 60%).

**Table 2:** Identified ORFs within phage HY01 genome and their BLASTp best hit. Only BLASTp matches with an E-value less than or equal to 0.0001 are listed.

ORF	Start	Stop	Strand	Length (bp)	BLASTp Best Hit (Gene) [Taxa]	Coverage (%)	E value	Identity (%)	Accession Blast Hit
1	1	603	+	603	small subunit terminase [Aeromonas phage phiARM81ld]	82	2e −31	40.48	ALN97521.1
2	683	2467	+	1785	large subunit terminase [Aeromonas phage phiARM81ld]	99	3e−120	38.47	ALN97522.1
3	2464	2673	+	210	head–tail joining protein [*Escherichia virus Lambda*]	95	1e−11	48.48	NP_040582.1
4	2673	4229	+	1557	Phage portal protein [Escherichia virus Lambda_2H10]	95	0.0	55.98	VUD36612.1
5	4222	5574	+	1353	Phage capsid and scaffold [*Escherichia virus Lambda*]	94	3e−121	45.90	VUF53141.1
6	5588	5929	+	342	Head decoration protein D [Aeromonas phage phiARM81ld]	72	2e−12	40.96	ALN97526.1
7	5969	7006	+	1038	phage major capsid E family protein [Escherichia phage YDC107_1]	98	6e−89	42.52	AUO37489.1
8	7071	7562	+	492	-	-	-	-	-
9	7571	7861	+	291	-	-	-	-	-
10	7858	8490	+	633	Prophage minor tail protein Z [*Escherichia virus N15*]	93	1.00e−44	41.62	NP_046906.1
11	8480	8929	+	450	tail protein [Enterobacteria phage mEp043 c-1]	95	4e−09	26.53	YP_007111512.1
12	8932	9429	+	498	major tail protein V [Enterobacteria phage HK225]	91	1e−47	51.66	YP_007112145.1
13	9432	9914	+	483	tail assembly chaperone [Enterobacteria phage phi80]	90	8e−05	27.52	YP_007947938.1
14	9938	10,270	+	333	Phage minor tail protein [Escherichia phage mEp460_ev081]	100	1e−05	35.14	VUF53221.1
15	10,251	12,473	+	2223	tail length tape-measure protein 1 [Pseudoalteromonas phage SL25]	68	8e−35	27.32	ASU03386.1
16	12,480	12,947	+	468	hypothetical protein [Pseudomonas phage PS-1]	74	1e−08	31.62	YP_009222842.1
17	12,947	14,743	+	1797	minor tail protein [Arthrobacter phage Ingrid]	48	2e−16	27.87	QFG08696.1
18	14,744	15,691	+	948	-	-	-	-	-
19	15,693	16,229	+	537	hypothetical protein [Bacteriophage sp.]	96	3e−28	40.70	QHJ79317.1
20	16,229	16,642	+	414	endopeptidase [Pseudomonas phage PS-1]	80	7e−08	32.43	YP_009222844.1
21	16,614	19,427	+	2814	tail protein [Pseudomonas phage PS-1]	98	4e−137	32.24	YP_009222845.1
22	19,471	19,731	+	261	hypothetical protein NVP1081O_260 [Vibrio phage 1.081.O._10N.286.52.C2]	89	9e−12	42.31	AUR85995.1
23	19,738	19,920	+	183	-	-	-	-	-
24	20,301	20,627	+	327	putative DNA-binding protein [Vibrio phage VHML]	73	5e−11	35.44	NP_758895.1
25	20,660	20,815	-	156	-	-	-	-	-
26	20,893	21,252	-	360	hypothetical protein CETO_181 [Vibrio phage Ceto]	89	4e−06	32.43	YP_009621244.1
27	21,272	21,562	-	291	-	-	-	-	-
28	21,583	21,813	-	231	-	-	-	-	-
29	21,824	22,786	-	963	ParB protein [*Escherichia virus N15*]	61	2e−51	46.70	NP_046922.1
30	22,798	23,187	+	390	-	-	-	-	-
31	23,252	23,377	+	126	-	-	-	-	-
32	23,594	23,731	+	138	-	-	-	-	-
33	23,733	23,817	+	85	tRNA-Ser-GCT	-	-	-	-
34	23,846	24,037	+	192	-	-	-	-	-
35	24,037	24,198	+	162	-	-	-	-	-
36	24,247	26,307	+	2061	protelomerase [Yersinia phage PY54]	74	3e−68	34.21	CAC88681.1
37	26,450	26,665	+	216	-	-	-	-	-
38	26,695	27,084	-	390	-	-	-	-	-
39	27,084	30,713	-	3630	primase [Aeromonas phage phiARM81ld]	84	0.0	36.02	ALN97565.1
40	30,768	30,971	-	204	-	-	-	-	-
41	31,075	31,770	-	696	prophage repressor [Enterobacterial phage mEp390]	62	7e−22	41.50	YP_007112454.1
42	31,912	32,139	+	228	putative transcriptional regulator (cro analog) [Salmonella phage Fels-1]	90	2e−04	36.23	YP_001700550.1
43	32,159	32,758	+	600	prophage antirepressor [*Halomonas virus HAP1*]	78	2e−16	25.77	YP_001686774.1
44	32,771	33,493	+	723	antiterminator Q [Yersinia phage PY54]	85	2e−12	26.42	NP_892088.1
45	33,698	34,393	+	696	-	-	-	-	-
46	34,424	35,101	+	678	hypothetical protein VH12019_00006 [Vibrio phage VH1_2019]	34	2e−10	34.62	QHJ74333.1
47	35,111	35,290	+	180	-	-	-	-	-
48	35,300	36,598	+	1299	putative exonuclease [Vibrio phage 1.205.O._10N.222.51.A7]	68	6e−116	54.73	AUR95306.1
49	36,598	37,104	+	507	single-stranded DNA-binding protein [Vibrio phage VD1]	100	7e−82	73.21	AGN34167.1
50	37,131	37,316	+	186	-	-	-	-	-
51	37,309	37,650	+	342	ASCH domain protein [Vibrio phage 1.104.O._10N.286.49.A12]	85	3e−06	35.00	AUR87783.1
52	37,650	39,077	+	1428	DNA methyltransferase [Vibrio phage Va_PF430-3_p42]	97	0.0	66.40	QCW19890.1
53	39,077	39,328	+	252	-	-	-	-	-
54	39,443	39,655	+	213	-	-	-	-	-
55	39,659	39,841	+	183	hypothetical protein VPQG_00007 [Vibrio phage VBpm10]	63	8e−11	68.42	AGF90980.1
56	39,845	40,093	+	249	-	-	-	-	-
57	40,093	40,476	+	384	hypothetical protein [*Escherichia virus N15*]	66	2e−18	42.86	NP_046953.1
58	40,493	40,621	+	129	-	-	-	-	-
59	40,835	41,116	+	282	Bacteriophage holin HP1 family protein [Bacteriophage APSE-7]	52	1e−07	46.94	CAB3623628.1
60	41,116	41,772	+	657	lysozyme-like domain protein [Vibrio phage 1.202.O._10N.222.45.E8]	97	4e−61	50.00	YP_009812536.1

Genes are listed by ORF numbers followed by their predicted function. - Represents no significant similarity found.

## Data Availability

The data presented in this study are available within the article and supplementary materials.

## Supplementary Materials

The following are available online at https://www.mdpi.com/2076-2607/9/2/400/s1: Table S1: Growth rate of *V. campbellii* HY01 after induction of prophage with mitomycin C, Table S2: Comparative genomics of phage and other related phages, Table S3: Summary of phage HY01genome.
