# Supplementary material for: Induction and Genome Analysis of HY01, a Newly Reported Prophage from an Emerging Shrimp Pathogen Vibrio campbellii
Source: Microorganisms. 2021 Feb 15;9(2):400. doi: 10.3390/microorganisms9020400 (PMC7919010; doi:10.3390/microorganisms9020400)
Supplement: Supplementary file 1 [file microorganisms-09-00400-s001.pdf]

Table S1. Growth rate (OD 600 nm) of *V. campbellii* HY01 after induction of prophage HY01 with mitomycin C.

Biological replicates	OD600 nm at time 0 h		OD600 nm at time 2 h		OD600 nm at time 4 h		OD600 nm at time 6 h		OD600 nm at time 12 h	
	***V. campbellii* HY01+mit C**	***V. campbellii* HY01**	***V. campbellii* HY01+mit C**	***V. campbellii* HY01**	***V. campbellii* HY01+mit C**	***V. campbellii* HY01**	***V. campbellii* HY01+mit C**	***V. campbellii* HY01**	***V. campbellii* HY01+mit C**	***V. campbellii* HY01**
1	0.297	0.329	0.289	0.544	0.187	0.637	0.338	0.734	0.675	0.858
2	0.404	0.332	0.314	0.484	0.290	0.589	0.345	0.713	0.772	0.863
3	0.399	0.364	0.286	0.496	0.253	0.606	0.333	0.690	0.792	0.917
mean	0.367	0.342	0.296	0.508	0.243	0.611	0.339	0.712	0.746	0.879
SD	0.060	0.019	0.015	0.032	0.052	0.024	0.006	0.022	0.063	0.033

Table S2. Comparative genomic of phage and other related phages.

Description	Max score	Total score	Coverage (%)	E value	Identity (%)	Accession
Vibrio phage Va_PF430-3_p42, complete genome	428	520	3	2e-115	70.68	MK672805.1
Pseudoalteromonas phage C5a, complete genome	300	300	1	7e-77	75.66	KY045851.1
Vibrio phage 1.205.O._10N.222.51.A7, partial genome	291	409	1	4e-74	78.53	MG592575.1
Vibrio phage 1.067.O._10N.261.52.C9, partial genome	208	208	1	4e-49	71.40	MG592444.1
Vibrio phage 1.003.O._10N.286.48.A2, partial genome	208	208	1	4e-49	71.40	MG592390.1
Vibrio phage 1.079.O._10N.286.45.E9, partial genome	196	311	1	2e-45	73.33	MG592454.1
Vibrio phage Rostov 7, complete genome	189	253	2	3e-43	70.98	MK575466.1
Vibrio phage X29, complete genome	184	269	1	1e-41	71.79	KJ572845.2
Vibrio phage 1.243.O._10N.261.54.B5, partial genome	178	178	1	6e-40	67.42	MG592608.1
Vibrio phage 1.106.O._10N.286.51.F7, partial genome	173	173	1	3e-38	67.88	MG592478.1
Vibrio phage V039C, complete genome	163	163	1	1e-35	67.45	MN956515.1
Vibrio phage 1.186.O._10N.286.49.E3, partial genome	162	162	1	5e-35	68.74	MG592552.1
Escherichia virus Lambda genome assembly, chromosome: 1	151	311	2	8e-32	65.61	LR597636.1
Vibrio phage phi 2, complete genome	150	236	1	8e-32	71.92	KJ545483.2
Escherichia virus Lambda_2B8 genome assembly, chromosome: 1	147	391	2	1e-30	66.80	LR595859.1
Escherichia virus Lambda genome assembly, chromosome: 1	138	314	2	5e-28	65.33	LR597653.1
Escherichia virus Lambda genome assembly, chromosome: 1	138	314	2	5e-28	65.33	LR597652.1
Escherichia virus Lambda genome assembly, chromosome: 1	138	314	2	5e-28	65.33	LR597651.1
Escherichia virus Lambda genome assembly, chromosome: 1	138	314	2	5e-28	65.33	LR597650.1
Escherichia virus Lambda genome assembly, chromosome: 1	138	314	2	5e-28	65.33	LR597648.1
Escherichia virus Lambda genome assembly, chromosome: 1	138	314	2	5e-28	65.33	LR597644.1
Escherichia virus Lambda genome assembly, chromosome: 1	138	377	2	5e-28	66.40	LR597639.1
Escherichia virus Lambda_2G7b genome assembly, chromosome: 1	138	380	2	5e-28	66.40	LR595866.1
Escherichia virus Lambda_2G7a genome assembly, chromosome: 1	138	314	2	5e-28	65.33	LR595865.1
Escherichia virus Lambda_4B5 genome assembly, chromosome: 1	138	314	2	5e-28	65.33	LR595863.1
Escherichia virus Lambda_2H10 genome assembly, chromosome:	138	368	2	5e-28	66.40	LR595862.1
Escherichia virus Lambda_2E9 genome assembly, chromosome: 1	138	314	2	5e-28	65.33	LR595860.1
Escherichia virus Lambda_1H12 genome assembly, chromosome: 1	138	314	2	5e-28	65.33	LR595850.1
Escherichia phage YDC107_1 chromosome, complete genome	138	314	2	5e-28	65.33	CP025712.1
Enterobacteria phage HK225, complete genome	122	275	2	4e-23	67.50	JQ086371.1
Enterobacteria phage phi80, complete genome	117	209	1	2e-21	67.25	JX871397.1
Enterobacteria phage phi80 partial sequence 5' end	117	209	1	2e-21	67.25	FN582354.1
Escherichia virus Lambda genome assembly, chromosome: 1	113	353	2	2e-20	66.67	LR597635.1
Enterobacteria phage mEp237, complete genome	113	205	1	2e-20	67.00	JQ182730.1
Bacteriophage CP-1639 and chromosomal integration site	109	170	1	3e-19	67.26	AJ304858.2
Bacteriophage N15, complete genome	107	442	2	9e-19	68.03	AF064539.1

Table S3. Summary of phage HY01genome.

Region	1
Region Length	41.7 Kb
Completeness(score)	Intact (110)
Specific Keyword	lysin, capsid, tail, head
Region Position	1-41772
# tRNA	0
# Total Proteins	60
# Phage Hit Proteins	34
# Hypothetical Proteins	26
Phage + Hypothetical Protein %	100%
# Bacterial Proteins	0
Attachment Site	No
# Phage Species	24
Most Common Phage Name (hit genes count)	PHAGE_Entero_N15_NC_001901(9)<br>PHAGE_Entero_DE3_NC_042057(8)<br>PHAGE_Entero_lambda_NC_001416(5)<br>PHAGE_Entero_HK225_NC_019717(5)<br>PHAGE_Yersin_PY54_NC_005069(5)<br>PHAGE_Pseudo_PMG1_NC_016765(4)<br>PHAGE_Escher_RCS47_NC_042128(4)<br>PHAGE_Halomo_phiHAP_1_NC_010342(4)<br>PHAGE_Vibrio_VP882_NC_009016(4)<br>PHAGE_Pseudo_phi297_NC_016762(4)<br>PHAGE_Entero_mEp237_NC_019704(4)<br>PHAGE_Vibrio_vB_VpaM_MAR_NC_019722(4)<br>PHAGE_Pseudo_D3_NC_002484(4)<br>PHAGE_Entero_HK630_NC_019723(4)<br>PHAGE_Pseudo_PS_1_NC_029066(4)<br>PHAGE_Klebsi_phiKO2_NC_005857(3)<br>PHAGE_Entero_phi80_NC_021190(3)<br>PHAGE_Entero_HK629_NC_019711(3)

PHAGE_Entero_P1_NC_005856(3)
PHAGE_Vibrio_VHML_NC_004456(3)
PHAGE_Vibrio_VP58.5_NC_027981(3)
PHAGE_Salmon_Fels_1_NC_010391(2)
PHAGE_Cronob_phiES15_NC_018454(2)
PHAGE_Pseudo_YMC11/07/P54_PAE_BP_NC_030909(2)
PHAGE_Pseudo_JBD44_NC_030929(2)
PHAGE_Pseudo_PAJU2_NC_011373(2)
PHAGE_Entero_c_1_NC_019706(2)
PHAGE_Entero_mEp213_NC_019720(2)
PHAGE_Entero_cdtI_NC_009514(2)
PHAGE_Vibrio_X29_NC_024369(2)
PHAGE_Escher_vB_EcoM_ep3_NC_025430(1)
PHAGE_Vibrio_Ceto_NC_042094(1)
PHAGE_Vibrio_KVP40_NC_005083(1)
PHAGE_Vibrio_12A4_NC_021068(1)
PHAGE_Aeromo_vB_AsaM_56_NC_019527(1)
PHAGE_Vibrio_12B8_NC_021073(1)
PHAGE_Vibrio_SHOU24_NC_023569(1)
PHAGE_Shewan_1/41_NC_025458(1)
PHAGE_Vibrio_VH7D_NC_023568(1)
PHAGE_Entero_mEp390_NC_019721(1)
PHAGE_Vibrio_8_NC_022747(1)
PHAGE_Vibrio_nt_1_NC_021529(1)
PHAGE_Vibrio_ValKK3_NC_028829(1)
PHAGE_Phage_Gifsy_1_NC_010392(1)
PHAGE_Vibrio_pVp_1_NC_019529(1)
PHAGE_Pseudo_MD8_NC_031091(1)
PHAGE_Vibrio_VvAW1_NC_020488(1)
PHAGE_Pectob_ZF40_NC_019522(1)
PHAGE_Vibrio_K139_NC_003313(1)
PHAGE_Vibrio_12B12_NC_021070(1)
PHAGE_Vibrio_pYD38_A_NC_021534(1)
PHAGE_Xantho_vB_XveM_DIBBI_NC_017981(1)
PHAGE_Pseudo_F116_NC_006552(1)
PHAGE_Pseudo_Pq0_NC_029100(1)
PHAGE_Pseudo_F10_NC_007805(1)
PHAGE_Entero_mEp460_NC_019716(1)
PHAGE_Salmon_SEN22_NC_028696(1)
PHAGE_Cellul_phi17:2_NC_021798(1)
PHAGE_Aeromo_pIS4_A_NC_042037(1)
PHAGE_Vibrio_SIO_2_NC_016567(1)

First Most Common Phage #	9
First Most Common Phage %	15%
GC %	47.45%

REGION: The number assigned to the region. REGION LENGTH: The length of the sequence of that region (in bp). PREDICT INTACT OR INCOMPLETE (score): A prediction of whether the region contains an intact or incomplete prophage based on the above criteria (with score in brackets). SPECIFIC KEYWORD: The specific phage-related keyword(s) found in protein name(s) in the region. REGION POSITION: The start and end positions of the region on the bacterial chromosome. TRNA NUMBER: The number of tRNA genes present in the region. TOTAL PROTEIN NUMBER: The number of ORFs present in the region. PHAGE HIT PROTEIN NUMBER: The number of proteins in the region with matches in the phage protein database. HYPOTHETICAL PROTEIN NUMBER: The number of hypothetical proteins in the region without a match in the database. PHAGE+HYPO_PROTEIN_PERCENTAGE: the combined percentage of phage proteins and hypothetical proteins in the region. BACTERIAL PROTEIN NUMBER: The number of proteins in the region with matches in the nrfilt database. ATT SITE SHOWUP: The putative phage attachment site. PHAGE SPECIES NUMBER: The number of different phages that have similar proteins to those in the region. MOST COMMON PHAGE NAME: The phage with the highest number of proteins most similar to those in the region. MOST COMMON PHAGE NUMBER: The number of phages with the highest number of proteins most similar to those in the region. MOST COMMON PHAGE PERCENTAGE: The percentage of proteins in PHAGE HIT PROTEIN NUMBER that are most similar to MOST COMMON PHAGE NAME proteins. GC PERCENTAGE: The percentage of gc nucleotides of the region.
